# ISEV position paper: extracellular vesicle RNA analysis and bioinformatics

**DOI:** 10.3402/jev.v2i0.22859

**Published:** 2013-12-23

**Authors:** Andrew F. Hill, D. Michiel Pegtel, Ulrike Lambertz, Tommaso Leonardi, Lorraine O'Driscoll, Stefano Pluchino, Dmitry Ter-Ovanesyan, Esther N.M. Nolte-‘t Hoen

**Affiliations:** 1 Department of Biochemistry and Molecular Biology, Bio21 Molecular Science and Biotechnology Institute, University of Melbourne, Parkville, Victoria, Australia; 2 Department of Pathology, VU University Medical Centre, Cancer Center Amsterdam, Amsterdam, The Netherlands; 3 Division of Infectious Diseases, Department of Medicine, University of British Columbia, Vancouver, Canada; 4 Department of Clinical Neurosciences, John van Geest Cambridge Centre for Brain Repair and Wellcome Trust-Medical Research Council Stem Cell Institute, University of Cambridge, Cambridge, UK; 5 EMBL – European Bioinformatics Institute, Wellcome Trust Genome Campus, Hinxton, Cambridge, UK; 6 School of Pharmacy and Pharmaceutical Sciences, Trinity College Dublin, Dublin, Ireland; 7 Department of Molecular and Cellular Biology, Harvard University, Cambridge, MA, USA; 8 Faculty of Veterinary Medicine, Department of Biochemistry and Cell Biology, Utrecht University, Utrecht, The Netherlands

**Keywords:** extracellular vesicles, deep sequencing, evRNA, RNA, bioinformatics

## Abstract

Extracellular vesicles (EVs) are the collective term for the various vesicles that are released by cells into the extracellular space. Such vesicles include exosomes and microvesicles, which vary by their size and/or protein and genetic cargo. With the discovery that EVs contain genetic material in the form of RNA (evRNA) has come the increased interest in these vesicles for their potential use as sources of disease biomarkers and potential therapeutic agents. Rapid developments in the availability of deep sequencing technologies have enabled the study of EV-related RNA in detail. In October 2012, the International Society for Extracellular Vesicles (ISEV) held a workshop on “evRNA analysis and bioinformatics.” Here, we report the conclusions of one of the roundtable discussions where we discussed evRNA analysis technologies and provide some guidelines to researchers in the field to consider when performing such analysis.

Extracellular vesicles (EVs) include exosomes and microvesicles that are released by many cell types into the extracellular space. Research in this field has increased dramatically over the last decade due to increased recognition of the role of EVs in cellular processes underlying homeostasis and disease pathobiology, and their potential use as biomarkers and therapeutic agents. A key discovery was that EVs contain RNA, allowing a role for these vesicles in the intercellular transfer of genetically encoded messages. The RNA types initially discovered in EVs were messenger RNA (mRNA) and microRNA (miRNA) (1,2). Currently, there is an increasing interest in analysing the complete transcriptome of EVs, and so far only a limited number of RNA deep sequencing based characterization of transcriptomes of different EV types have been published (3–6). With the rapid advances in deep sequencing or Next Generation Sequencing (NGS) techniques, large numbers of unexpected non-coding RNA species have been discovered in cellular transcriptomes (7). These transcripts were found to overlap with exons, introns, and intergenic regions, (8) and several of the newly discovered non-coding RNAs have been implicated in regulation of transcription or translation. Interestingly, EVs were found to be enriched in several types of such non-coding RNA species (3–6). Currently, the mechanism(s) responsible for extracellular vesicle RNA (evRNA) trafficking and release, in addition to the function of this RNA identified in a heterogeneous pool of EVs, remains unclear. Since most of the transcriptome undergoes extensive post-transcriptional modification, this may also add to the complexity of evRNA. Given the additional complexities concerning the nomenclature of EVs, isolation methods, variety of platforms for deep sequencing and downstream bioinformatics analysis, the topic of “evRNA analysis and bioinformatics” was discussed at the International Society of Extracellular Vesicles (ISEV) Research Seminar held in New York in October 2012. Here, we present a summary of the roundtable discussion of this topic and present points of consideration and awareness to researchers working in the area of evRNA analysis, in addition to defining the challenges associated with this area of research. This position paper should be read in conjunction with the recent publication by Witwer et al. in this journal (9) that covers the discussion on sample collection, vesicle isolation, and analysis methods in EV research.

## Source of material for evRNA analysis

Vesicles can be isolated from a number of sources including cell culture supernatants and biological fluids, such as plasma, serum, urine, milk, and cerebrospinal fluid. Different methods may be required for effective isolation of EVs from these different sources (9). With regard to parameters influencing evRNA analysis, we discuss below some of the key issues for EVs isolated from cell culture supernatants and bodily fluids.

### Cell culture supernatants

Many studies utilize cell culture supernatants as a source of EVs for analysis. We considered several experimental variables that should be reported when presenting analysis of evRNA. Reporting the confluency and viability of the cells using commercially available reagents would allow assessment of the culture health prior to evRNA analysis. Apoptotic or necrotic cells in cell cultures can release RNA-containing apoptotic bodies or nucleoprotein complexes, which contaminate the evRNA population. Ideally, a control experiment is to induce apoptosis in a culture and prepare EVs (10), comparing their properties with those of the experimental sample. Furthermore, as many culture systems can be contaminated with mycoplasma, a statement as to whether the cultures used in the analysis were checked for the presence of these contaminants should be provided.

The method used to isolate the EVs should be provided in as much detail as possible, allowing exact replication of the approach. Descriptions should include the number of cells cultured, volumes of media, centrifugation parameters (including rotor and tube type, centrifugal force, time), and the downstream storage conditions prior to RNA extraction. Fractionation of pelleted vesicles using density gradient media (such as sucrose or iodixanol) is recommended to isolate intact vesicles separated from contaminants.

With regard to the culturing conditions, it is important to detail whether vesicle-containing supplements, such as foetal calf serum (FCS) were added to the culture medium and how EVs were depleted from these additives before use. Furthermore, characterization of the isolated vesicles should be performed to demonstrate the presence of vesicles and provide an assessment of their purity. Techniques such as transmission electron microscopy (TEM) (11), nanoparticle tracking analysis (NTA; Nanosight) (12) and tunable resistive pulse sensing (qTRS; qNano) (13) can be employed to provide analysis of the approximate size of vesicles present in the preparation. Additionally, flow cytometry, NTA, qNano, and dynamic light scattering (DLS) have been used to provide quantitative data for EVs (14–17). Finally, the detection of various protein markers commonly found in EVs (18) can be carried out to further demonstrate the type of vesicles isolated (9).

### Body fluids

Methods used for the collection of body fluids and storage procedures may largely influence the range of EVs isolated from these fluids [see (9) for a detailed description], and thereby the RNA profile of the body fluid contained EVs. The method of choice for EVs isolation from different body fluids may depend not only on the type of body fluid, but also on the volume available, and parameters such as the viscosity of the fluid. As with the isolation procedures for cell culture derived EVs, publications should include a clear explanation of the steps used (e.g. centrifugation parameters) to obtain and characterize the EVs from body fluids. It is also recommended to establish a working protocol for the isolation of EVs and subsequently RNA before processing of precious samples. Parameters such as the delay in processing samples, storage temperatures, and the available volume of samples all contribute to the yield of EVs from which RNA is extracted. Confirmation of the presence of EVs in the sample, using the methods described above for cell culture supernatants, is also necessary.

While sample collection procedures can be standardized to a certain degree, there will also be inherent variablities in the samples that are more pronounced than when using cell supernatants, which can be controlled more readily. Proposed checklists for sample choice and sample collection/processing are presented in detail in Witwer et al. (9).

## Treatment of samples with nuclease – or not?

Besides RNA associated to EVs, biological samples also contain RNA species that may be bound to other molecules such as proteins and lipid complexes (19–21). EvRNA should be protected from nucleases activity in the environment by the EV lipid bilayer. RNase treatment of EVs has therefore been used to discriminate evRNA species from extracellular RNA not associated to EVs. However, it should be noted that several body fluids (e.g. saliva milk and urine) contain (high levels of) nucleases, making the presence of RNase-sensitive complexed RNAs in such fluids unlikely (22). Additionally, proteins on the surface of EVs may bind and protect RNA from degradation, hence the addition of exogenous RNase tends to be ineffective in the absence of proteases. It is currently unknown whether both nucleoprotein complexes on the outside of EVs and luminal evRNA can be functionally delivered to target cells. Nevertheless, RNase treatment could be effective in removing contaminating RNA molecules passively released by dead cells that may non-specifically stick to EVs in environments that are low in nuclease content. Importantly, nuclease levels in body fluids can change due to pathological conditions. In cancer patients, for example, increased levels of serum nucleases have been observed (23). Such variations in nuclease levels should be taken into account when analysing evRNA present in clinical samples. Nevertheless, RNase treatment could be effective in removing contaminating RNA molecules passively released by dead cells that may non-specifically stick to EV's in environments that are low in nuclease content. The use of external spike-in RNA controls, to test whether the EV-containing fluid being analysed contains endogenous RNase activity, was discussed and considered to be unnecessary as the input material would be degraded almost immediately, precluding downstream detection (24).

RNaseA, which is specific for single-stranded RNA, has been mostly used in evRNA analysis. A potential risk in treating EV samples with RNase is that high concentrations of RNases are difficult to inhibit and residual activity may affect the yields of RNA isolated from the sample. The extent to which lysis buffers, used in the first step of the evRNA isolation procedure, can control such activity is unknown. In case RNase treatment of EVs is applied, the effectiveness of the RNase treatment should be assessed by combined treatment with detergents, which should lead to more efficient and complete degradation of the evRNA. Whether to treat a particular sample with RNase or not depends on the research question being asked. For instance, if the purpose of the experiment is to examine how RNAs are selectively incorporated into EV biogenesis, treatment with RNase may be essential.

Besides the presence of extracellular RNA, some biological fluids are rich in DNA (for instance, plasma). It should be noted that none of the available RNA extraction methods excludes co-isolation of DNA entirely, and that contaminating DNA may interfere in RNA Bioanalyzer profiling and deep sequencing. It is therefore advisable to treat samples suspected of DNA contamination with DNase prior to evRNA isolation.

## Considerations of the number of replicates

Another consideration when performing RNA deep sequencing experiments is the number of replicates, which can be either technical or biological in nature. The first step of deep sequencing protocols is preparing the library to be sequenced. In this step of the procedure some variation may be introduced, depending on the protocol and platform. The protocols for addition of adapters as part of the library preparation vary between the sequencing platform employed; the adapter sequences vary widely and may introduce bias, which needs to be assessed (see paragraph on library preparation). The use of a second method for RNA detection is employed to preferably validate the sequencing results (see below for more details). The sequencing of the libraries on the other hand seems highly reproducible. Repeated sequencing of the same library is therefore not required, unless for increasing the depth of sequence coverage. Increasing the depth of sequence coverage not only allows for the detection of rare transcripts, it may also identify sequences of other origins such as those from bacterial and viral sources. In addition to the sources of technical variability listed above (i.e. library preparation and sequencing), biological variability must also be estimated and taken into account in order to detect significant differences in RNA abundance. For this reason, including biological replicates in the experimental design is critical in order to obtain biologically meaningful results (25,26). In the event that deep sequencing of evRNA in patient samples is used for diagnostics, emphasis should be put on defining biofluid-specific normatives and appropriate control groups (9). Although small RNA sequences are thought to have low genetic variability (27), the extent to which small RNA expression levels vary between individuals has not been fully clarified. The choice to either pool patient samples or perform RNA sequencing on individual samples will also be dependent on the obtainable amount of RNA and financial resources.

## Microarray analysis of RNA from EVs

While the discussion at the roundtable mainly focussed on the use of deep sequencing approaches for the analysis of EV RNA, microarray technology has also been used to study this RNA. Microarrays are a well-established technique for profiling the expression of known fragments of nucleic acids using slide or chip-based media. Microarrays are available for screening mRNA, miRNA, long non-coding RNA (lncRNA) species and offer an attractive advantage in that they do not rely on the use of specialized sequencing equipment and/or complex bioinformatics approaches (discussed below). However, as microarrays use only known sequences as targets, the ability to detect potentially novel sequences is therefore not possible. Moreover, microarrays entail a risk for probe cross-hybridization and, in contrast to NGS, do not directly count the number of transcripts (28). Also, given the updates to databases such as miRBase (currently at version 20), microarrays need to be updated in line with these. Nevertheless, microarrays present a useful tool for profiling EV RNA species and have been used in studies of both EVs from cell culture systems and those isolated from bodily fluids (16,29,30).

## Deep sequencing platforms

Over the last couple of years, several technologies have been developed for deep sequencing. Many rely on proprietary kits and equipment for performing deep sequencing. Presently, the major systems in use are the Illumina HiSeq, Roche 454 pyrosequencing, and the SOLiD system from Applied Biosystems. These platforms are generally used in high-coverage sequencing projects and operated through service providers. Recently, smaller deep sequencing platforms have become available, which allow individual laboratories to perform their own sequencing. These include the MiSeq (Illumina), Ion Torrent Personal Genome Machine (Life Technologies), and the GS Junior (Roche). One consideration when choosing a sequencing platform to use, or decide between the personal or larger machines, is the depth of coverage obtained with the particular system. Deeper coverage will allow the analysis of rare sequences and give more confidence in the number of reads per transcript. However, there are cost and time constraints, which also have to be considered.

## Library preparations from evRNA samples

Although the majority of RNA in various types of EVs consists of small RNAs (20–200 nt) (1,3,4,31,32), EVs can also contain larger RNAs, such as mRNAs and low levels of 18/28S ribosomal RNA (1,33). Sequencing of small and large RNAs requires different steps in the sequencing library preparation protocol. A comparison of commonly used library preparation methods is given in Table I. Since the small RNA pool in EVs can contain small transcripts or cleavage products overlapping with larger (non)coding RNAs, fragmentation of total RNA isolates before library preparation can lead to erroneous interpretation of the small evRNA profile. In general, procedures should be carefully considered to ensure valid interpretation and comparative analysis of sequencing data. We have listed important points of consideration in Table II.

**Table I:** Small-RNA-sequencing library preparation: most widely used library construction methods

Method	Step 1	Step 2	Step 3	Step 4
*Hybridization method (e.g. SREK for SOLiD)*	Two double-stranded adapters that contain degenerate 5′- or 3′-end overhangs	Ligation adapters with T4 RNA ligase	Reverse transcription into cDNA	Further amplification by PCR using primers that anneal to both adapter sequences
*Polyadenylation based (A)*	Addition 3′ poly A tail using Poly (A) polymerase	Ligation RNA adapter to the 5′ end using T4 RNA ligase	Reverse transcription into cDNA	Further amplification by PCR
*Polyadenylation based (B)*	Addition 3′ poly A tail using Poly (A) polymerase	Reverse transcription into cDNA	Ligation adapter to the 5′ end using T4 RNA ligase 1	Further amplification by PCR
*Sequential adapter ligation (e.g. Illumina) (A)*	Sequential ligation of 3′- and 5′-adapter oligonucleotides directly to small RNA	Ligation RNA adapter to the 5′ end using T4 RNA ligase	Reverse transcription into cDNA	Further amplification by PCR
*Sequential adapter ligation (e.g. Illumina) (B)*	Sequential ligation of 3′- and 5′-adapter oligonucleotides directly to small RNA	Reverse transcription into cDNA	Ligation adapter to the 5′ end using T4 RNA ligase 1	Further amplification by PCR

**Table II:** Parameters with regard to sRNA library preparation that should be considered

Parameter	Effects to be considered	Approaches
RNA structures	Some 5′- or 3′-end modifications of RNA sequences are not reactive or have reduced reactivity for enzymatic steps (adapter ligations) involved in library preparation. Sequences of small RNAs and their secondary and tertiary self-structures can affect the efficiency of 3′- and 5′-adapter ligation (34).	Enzymatic treatments can be used to convert small RNAs of interest to have appropriate and homogenous ends in order to be ligated to adapters with good efficiency (35).
RNA size distribution	Potential transcript length bias	Selection of small RNAs of specific sizes increases sequencing depth for sequences of interest.
Library construction method	Different barcode sequences can influence the RNA and adapter cofold structures, which may cause changes in ligation efficiency (36) Occurrence of adapter dimers	Use barcodes positioned in primers during library pre-amplification (37) or post-amplification barcode strategies (38). Removal of adapter dimers improves sequencing of specific products; removal of non-productive sequencing reads.

Prior to library preparation, the evRNA size distribution should be analysed, e.g. by Bioanalyzer profiling, and these data should be included in publications. It should be noted that RNA Integrity Numbers (RIN), provided by the Bioanalyzer software, are not indicative of the integrity of small evRNA, since RIN are based on the major 18S and 28S ribosomal RNA peaks that are abundantly present in cells but not in most EV types.

Small RNAs can have different modifications at the 5′- and 3′-termini due to their diverse origins and biogenesis pathways. Some of these modifications are either not reactive or less reactive to enzymatic manipulations in the expression profiling protocols, such as adapter ligation by T4 RNA ligase (35). Treatments of the RNA pool to be sequenced with enzymes such as phosphatases can be applied to enrich for or exclude classes of RNAs in sequencing libraries (35).

The most widely used library construction methods are listed in Table I. The library preparation generally involves adapter ligation to both ends of the RNAs, reverse transcription into cDNA and further amplification using primers specifically annealing to the adapter regions. It should be noted that biases might be introduced during library preparation that can affect the quantitative aspect of the expression profiling. These biases include differences in the efficiency of adapter ligation to certain RNAs due to the T4 ligase or differences in the barcode sequences (in case of multiplex sequencing) and biases introduced during polymerase chain reaction (PCR) amplification. Validation experiments using different techniques can be used to confirm relative expression differences between samples (see below).

The adapter-ligated cDNA can be run on a gel, after which bands can be excised to exclude adapters without inserts or to select for cDNAs in the preferred size range. Images of the selected bands (with sizes) should be supplemented in the publication, and it is advisable to include a Bioanalyzer profile of the library. This latter step, however, can cause substantial variation between experiments. Although size selection may improve the sequencing of specific products, the depth of sequencing that can currently be achieved by the advanced deep sequencing techniques is usually sufficient to be able to detect rare products.

With regard to data comparability, the use of identical protocols for library preparation is crucial for valid and reproducible comparison of RNA levels between different libraries. Nevertheless, external spike-in RNA controls can be useful to evaluate the sensitivity, accuracy, and comparability of RNA-sequencing experiments (39).

## Bioinformatics and databases

Deep sequencing of evRNA generates massive amounts of data, which need to be analysed using appropriate bioinformatics tools. Quality metrics of the sequenced reads – such as per-cycle quality score, nucleotide frequency, and read complexity – give an important initial estimate of the quality of the data. The alignment of sequence data back to reference genome is a critical step in the bioinformatic analysis of deep sequencing data. Low-percentage alignment of sequencing reads against the reference may indicate low quality or abundance from the starting material. It is important to include the alignment parameters (e.g. % alignment, database/reference genome to which the data were aligned to) in publications containing the RNA-sequencing data. Care should also be taken when choosing what method to use to normalize the data. Common normalization strategies are based on the total number of mapped reads, the total number of reads mapping to the features of interest (e.g. total number of reads mapping to miRNAs), or spike-ins. Since a standard consensus strategy is still lacking in the field, ideally more than a single normalization methods should be adopted to show the consistency of the results. Among a number of possible approaches, the two most likely strategies that emerged were to normalize over either the total number of mapped reads or the number of reads mapping to miRNAs. Each of the two approaches has its advantages, and the choice depends on what comparison one is trying to make.

Furthermore, details as to the pipeline of data analysis steps from sequencing to compiling the final results are required. As many steps as is practicable should be described in the publication, so that other researchers can perform similar analysis using deep sequencing data from other EV samples. This metadata will allow the analysis of variability due to experimental conditions and processing parameters across different studies. This should include the processing steps applied to the raw data, any details of trimming/clipping applied to the reads, criteria for cut-off values, the databases used in the analysis, and the order in which these steps were performed on the data. The version numbers of any bioinformatics tools or databases interrogated also need to be provided as these are constantly being developed and updated.

As the field evolves to include sequence data from EVs of different sources, the sharing of data is essential; both as a reference tool and also with confirming previously deposited data. It is important to deposit data sets in relevant depositories including NCBI, and also in EV-specific databases, including Vesiclepedia (40) and EVpedia (41). The parameters described above should also be deposited with the sequencing data.

## Validation of deep sequencing data

Validating deep sequencing data arising from EV samples with a different technique is important for a number of reasons. Firstly, the preparation of sequencing libraries and platforms differs between providers. Secondly, validating data sets is important for standardization between different laboratories. The most commonly employed validation technique is quantitative real-time PCR (qPCR). Validation with qPCR also allows the screening of more biological replicates without the need for deep sequencing of many samples. The choice of a normalization control is important when comparing expression levels between replicates and treatment conditions. It should be noted that those household genes that are generally used to normalize expression levels between samples of cellular RNA are not per se appropriate normalization controls in samples of evRNA. The reliability of applied evRNA normalization controls should be indisputably demonstrated by showing proof that incorporation levels of such control RNAs into EVs are steady in each of the experimental conditions tested. In fact, where possible, it is advisable to use multiple reference genes for qPCR data normalization. A far more feasible approach is to perform a global normalization to quantitative data, in which mean expression values of all expressed small RNAs are used for data normalization (42). Using this method, the ratio of two particular miRNAs in the deep sequencing data could, for example, be compared to the detected ratio using qPCR. The advent of digital PCR techniques with which the exact number of target molecules in a sample can be assessed may assist in finding appropriate normalization controls for each experiment.

## Conclusions and perspectives

In-depth analysis of evRNA using advanced sequencing techniques can yield valuable information on how genetically encoded messages are exchanged between cells. However, methods that can be used for EV isolation, evRNA isolation, sequencing library preparation, as well as sequencing data analysis are highly diverse. To ensure well-considered experimental setups and to allow for inter-laboratory comparisons, it is important to comprehend parameters that can influence experimental outcomes and include highly detailed descriptions of experimental layouts in scientific publications. This also allows for the sharing of knowledge that is often not in the public domain, but which allows the evRNA field to move forward more rapidly. Our current position statement is based on the roundtable discussion on “evRNA analysis and bioinformatics” during the ISEV workshop on RNA in EVs, and provides points of consideration in setting up evRNA sequencing analysis. In addition, we recommend the specification of as many details as practicable in publications containing such analysis. A checklist for these details is provided in Table III. It is important to provide all details on the RNA isolation and purification methods used and RNA quality control experiments performed. For the deep sequencing, the library preparation conditions need to be specified in terms of the quantity of input RNA, kit used (including the version, as these kits are continuously under improvement), and quality control of the library itself. Furthermore, it is important to report the sequencing conditions and to provide quality scores (Q scores), depth of coverage, and number of reads. Also with regard to the bioinformatics analysis of the sequencing data, full details on processing steps, criteria, and databases used should be reported. Posting the raw data to one of the major databases [e.g. Sequence Read Archive (SRA), Gene Expression Omnibus (GEO), Vesiclepedia (www.microvesicles.org) and EVpedia (www.evpedia.info)] is also essential, and often a requirement of publishing, so that other researchers can assess the data. In conclusion, the points of awareness and consideration raised in this paper should help researchers in the field of evRNA to critically design and control their experiments and should facilitate inter-laboratory comparison of evRNA data sets.

**Table III:** Checklist experimental details for publication

Step	Parameters to be described
Cell culture	Cell type Confluency Use of vesicle-depleted serum or serum-free medium Cell viability Mycoplasma test
EV isolation	Differential centrifugation steps Filtration steps Density gradient Commercially available kits (e.g. chemical, column based)
evRNA sample preparation	RNase/DNase treatment of EVs Use of RNA spike RNA isolation method/kit Bioanalyzer profile of evRNA
Library preparation	Enzymatic treatment to remove phosphates, caps, etc. Method/kit for ligating adapters Pre-amplification steps Size selection steps Bioanalyzer profile of library
Sequencing	Platform Maximum read length Direction (paired or single end) Number of cycles Number of replicates (biological or technical)
Bioinformatics	Pre-processing software (trimming/clipping, cut-off values) Reference genome assemblies (release numbers) Primary analysis software (alignment and mapping criteria) Browsers and annotation tools Normalization methods and software Statistical methods and tools for differential expression analysis
Validation	Validation technique Normalization procedure
Deposition in database	Name of database
